# The clinical and demographical profile of Coronavirus illness: The tale of *Tablighi Jamaat* and *Zaireen* in Quarantine / Isolation center at Sukkur and Hyderabad

**DOI:** 10.12669/pjms.36.COVID19-S4.2829

**Published:** 2020-05

**Authors:** Ikram Din Ujjan, Bikha Ram Devrajani, Akbar Ali Ghanghro, Syed Zulfiquar Ali Shah

**Affiliations:** 1 Prof. Ikram Din Ujjan, Department of Pathology, LUMHS, Jamshoro, Pakistan; 2 Prof. Bikha Ram Devrajani Department of Medicine, LUMHS, Jamshoro, Pakistan; 3 Akbar Ali Ghanghro Field Epidemiologist / National Stop Transmission of Polio Program in Pakistan, (CDC-USA, WHO and MOH funded program) LUMHS, Jamshoro, Pakistan; 4 Syed Zulfiquar Ali Shah Assistant Professor, Department of Medicine LUMHS, Jamshoro, Pakistan

**Keywords:** Corona, COVID-19 and SARS CoV2, Virus

## Abstract

**Objectives:**

To determine the clinical and demographical profile of corona-virus illness among *Tablighi Jamaat and Zaireen* kept in quarantine / isolation center at Sukkur and Hyderabad Sindh.

**Methods:**

The cross-sectional descriptive study (late March-2020 to mid of April-2020) was conducted at Diagnostic & Research Laboratory LUMHS Jamshoro / Hyderabad. All the suspected cases for COVID-19 were recruited and screened for corona virus infection. The study explored the data of the suspected and diagnosed (confirmed) case of COVID-2019 (Tablighi Jamaat and Zaireen) reported by Diagnostic Research Laboratory Liaquat University of Medical and Health Sciences (LUMHS) Jamshoro who belonged to various parts of the country in general and province Sindh in particular. All the individuals regardless of age and gender presented either as asymptomatic, critical ill or having non-specific symptoms as fever, flu, cough; sore throat and shortness of breath were screened for COVID-19 by real time PCR after taking informed consent whereas the frequency / percentages (%) and means ±SD computed for study variables.

**Results:**

During study period total 920 patients were explored and screened for Corona virus infection. The mean ± SD for age (yrs) of overall population of city Sukkur and Hyderabad was 57.83±8.84 and 59.62±9.72 respectively. The 700 people from Sukkur city was screened and out of them 276 (39.4%) were positive and 424 (60.5) were negative while the cure rate was 245 (88.7%) along with mean ± SD for recovery time was 9.41±2.97. The 220 people from Hyderabad city was screened and out of them 106 (48.1%) were positive and 114 (51.8%) were negative while the cure rate was 106 (100%) along with mean ± SD for recovery time was 11.54±3.42. The majority of cases at both centers were asymptomatic (90%), symptomatic (7%) and critically ill (3%). The mortality accounted for 2.8% cases at Hyderabad isolation center and all were having smoking history and co-morbidities as ischemic heart diseases, diabetes mellitus, obstructive lung disease and cerebrovascular accident whereas no mortality was observed at Sukkur isolation center.

**Conclusion:**

RT-PCR measure allowed fast, delicate, and explicit discovery of SARS-CoV in biochemical diagnosis. The majority of cases at both centers were asymptomatic while the mortality was identified in 2.8% cases (having co-morbidities) at Hyderabad isolation center whereas no mortality was observed at Sukkur isolation center.

## INTRODUCTION

Coronavirus illness or generally known as COVID-19 is the updates on each and every second since it began from Wuhan, China, which is announced as a pandemic disease by World Health Organization, said to be brought about by another strain of the infection.^1^ The illness is said to spread through droplets of saliva or discharge from the nose of a contaminated individual, especially when he/she sniffles or coughs.^2^ In spite of the fact that the human coronaviruses have been perceived now for a long time, the absence of invulnerability to the recognized new strain, there is a huge segment of the population vulnerable to it. Furthermore, the most recent pattern of the exponential increment in the tainted individuals, with a precarious ascent of 69.17% within three days from 21^st^ March 2020 to 23^rd^ March 2020 and in light of the absence of preventive immunization, there has been a high fearful situation amongst the local public.^3^,^4^ The disease severity has varied from self-restricting influenza like illness to fulminant pneumonia, respiratory embarrassment and mortality. There are variations in the death rates and these assessments are quickly changing as more information is opening up and in process.^5^ As per WHO, Coronavirus disease (COVID-19) Situation Report – 106 released on May 6, 2020, total confirmed cases of COVID19 globally are 3, 517, 345 and total Deaths were 2,43, 401 .^6^

A much lower mortality of 1.4% has been accounted for exploration of data of 1099 patients with research center affirmed COVID-19 from 552 clinics in mainland China.^7^ Since the start of the coronavirus pandemic there has been an extended use of spreads and sanitizers realizing exhaustion of resources in the market. An inadequacy of individual guarded apparatus endangers prosperity workers around the globe.^8^ The non availability of appropriate protective measures like PPEs, proper N95 masks and other protective measures is a matter of concern among hospital workforce.The problem is acute in a country like Pakistan which is a thickly populated without a strong and well established health infrastructure.^9^ Public has been advised through mass media against mass gatherings of all sort including sports, schools and religious nature to heck the spread of this deadly virus. In spite of these endeavors, numerous individuals overlook the significance of social isolation because of public attitude.^10^-^12^ The development of SARS-CoV-2 has once again exposed the weaknesses of global health systems preparedness, capacity to react to an irresistible danger, the rate of transmission of contaminations across universal borders and the insufficiency of proper arrangement to counter the rising/reappearing irresistible infection threat.^13^-^15^

Taking into account that the confirmed cases the comprehension of epidemiological attributes of this contamination is advancing every day, the infection is spreading to various parts of the globe presented either with symptomatic or critically ill or remain asymptomatic. This audit can provide important data to future research and may bolster government dynamics on systems to handle this public health emergency at the national and international level.

## METHODS

The descriptive case seriesstudy explored the data of the suspected and diagnosed (confirmed) case of COVID-2019 during late March-2020 to Mid of April-2020 reported by Diagnostic Research Laboratory Liaquat University of Medical and Health Sciences (LUMHS) Jamshoro of patient who belonged to various parts of the country in general and province Sindh in particular. The population were divided in two parts i.e. Tablighi Jamaat and Zaireen, the former group were kept in isolation center at Hyderabad and had history of contact exposure since they had started their religious journey from Raiwind & have Hyderabad visit as an religious meeting & gatherings while the latter group was kept in isolation center at Sukkur and had history to visit religious place (pilgrims) at Iran and came through Taftan border to the Sindh province. The peoples of *Tablighi Jamaat* group belonged to various cities of Pakistan while the *Zaireen* group were from various cities of Sindh province. All the cases regardless of age and gender presented either as asymptomatic, critical illness or having non-specific symptoms as fever, flu, cough; sore throat and shortness of breath were then screened for COVID-19 by real time PCR after taking informed consent. From the point that a person is tested, a sample has been taken after all aseptic measure by trained technician under the supervision of health care provider and then transported carefully to a diagnostic & research laboratory LUMHS and analyzed through the RNA extraction done by Abbott RT auto extraction system by using extraction kits of Abbott Labs. and detection was done on ABI thermal cycle by haploid bio system by using sansure detection kits. The study was conducted after the approval of ethical review committee LUMHS and the data was collected on pre-designed proforma and analyzed in SPSS-22 to manipulate the frequencies / percentages and mean ± SD of categorical as well as numerical variables.

## RESULTS

During the study period total 920 patients were explored and screened for Corona virus infection. The mean ± SD for age (yrs) of overall population was 57.83±8.84. The demographical and clinical profile of study population (Sukkur and Hyderabad) quarantine / isolation centers is presented in Table I and II. The majority of cases of both centers were asymptomatic (90%), symptomatic (7%) and critically ill (3%). The mortality was seen in 2.8% cases of Hyderabad isolation center and those who died had smoking history and also co-morbidities as ischemic heart diseases, diabetes mellitus, obstructive lung disease and cerebrovascular accident whereas no mortality was observed at Sukkur isolation center.

**Table-I:** The clinical and demographical profile of Quarantine / Isolation center at Sukkur.

Parameter	Frequency (N=700)	Percentage (%)
CITY:		
SUKKUR		
Positive	276	39.4
Negative	424	60.5
GENDER		
Male	147	52.8
Female	129	46.7
PILGRIM TRAVEL HISTORY	276	
RT-PCR STATUS		
0 (PCR positive)	276	
7^th^ day (First PCR negative)	178	64.4
After 24 hours (second PCR still positive)	98	35.5
14^th^ day (Second PCR negative)	67	68.3
After 24 hours (second PCR positive)	31	31.6
CLINICAL STATUS:		
Cured / Recovered	245	88.7
Remain positive PCR	31	11.2
AVERAGE RECOVERY TIME (mean ± SD)	9.41±2.97	

**Table-II:** The clinical and demographical profile of Quarantine / Isolation center at Hyderabad.

Parameter	Frequency (N=220)	Percentage (%)
CITY:		
HYDERABAD		
Positive	106	48.1
Negative	114	51.8
GENDER		
Male	106	52.8
PILGRIM TRAVEL HISTORY	220	
RT-PCR NEGATIVE		
0 (PCR positive)	106	48.1
7^th^ day(First PCR negative)	101	95.2
After 24 hours (second PCR still positive)	05	4.7
14^th^ day (second PCR negative)	02	100
After 24 hours (second PCR negative)	02	100
CLINICAL STATUS:		
Cured / Recovered	103	100
Remain positive PCR	-	-
Mortality	03	2.8%
AVERAGE RECOVERY TIME (mean ± SD)	11.54±3.42	-

## DISCUSSION

COVID-19 pneumonia broke out in Wuhan, on January 30^th^, 2020 and the pneumonia scourge brought about by a novel coronavirus was declared an emergency worldwide by the WHO.^16^ The wellspring of the contamination was a novel coronavirus (SARS CoV2). Until now, respiratory beads and direct contact have been recognized as the primary transmissions routes.^17^ Vaporized and gut related transmission has still to be confirmed. The incubation duration of the disease is commonly 3-7 days, however no longer than 14 days. Because of its solid infectivity profile, early diagnosis and treatment are critical; in any case human intervened spread can seriously imperil general population. In intense respiratory disease, RT-PCR is routinely used to identify causative infections from respiratory emissions.^19^

The current study depicts the foundation of a symptomatic work process for existence of a developing infection without physical sources of viral genomic nucleic acid. Powerful examine configuration was empowered by the ability of researchers from China to share genome data before formal distribution, just as the accessibility of expansive succession information.^20^ The relative straightforwardness with which tests could be performed for this infection, rather than SARS-CoV in 2003, demonstrates the gigantic aggregate estimation of elucidating investigations of illness nature and viral genome decent variety.^21^ Real time (RT-PCR) test is generally recommended in virology. On account of a general population emergency, capable demonstrative research facilities can depend on this hearty innovation to build up new symptomatic tests inside their standard administrations before pre-formulated assays become accessible.^22^ Notwithstanding data on reagents, oligo-nucleotides and positive controls, research facilities working under quality control programs need to depend on documentation of specialized capability of the assay plan just as information from clinical assessment tests.^23^ This laboratory limit bolsters quick general public health exploration well as empowers destinations to enlist patients during rapid laboratory diagnosis. In the study by Mizumoto K et al.^24^, of the 634 diagnosed cases, a total of 306 and 328 were observed to be symptomatic and asymptomatic and soon after detection of infections both groups were transported to get the medical facilities at hospitals. Indeed the asymptomatic individuals are useful quantity to determine the true burden of disease & can better interpret the transmission burden as evidence highlights that the fraction of SARS-CoV-2 positive individuals are mostly asymptomatic.^25^

## CONCLUSION

RT-PCR measure allowed fast, delicate, and explicit discovery of SARS-CoV in biochemical diagnosis and offered required indicative help during the ongoing outbreak. Broadly speaking this test will improve our capacity to go for a quick detection in case of the conceivable return of SARS-CoV.The majority of cases of both centers were asymptomatic (90%), symptomatic (7%) and critical ill (3%). The mortality was seen in 2.8% cases at Hyderabad isolation center and all were having smoking history and co-morbidities as ischemic heart diseases, diabetes mellitus, obstructive lung disease and cerebrovascular accident whereas no mortality was observed at Sukkur isolation center.

### Authors Contribution

**IU:** Diagnostic testing, reviewing manuscript, study supervision and acquisition of data

**BRD:** Conception, design of the study, review of manuscript d & final approval of version to be published.

**AAG:** Data collection and its analysis.

**SZAH:** Interpretation of data,, manuscript editing, supervision of the study.
